# Microenvironmental Modulation for Therapeutic Efficacy of Extracellular Vesicles

**DOI:** 10.1002/advs.202503027

**Published:** 2025-03-27

**Authors:** Bilu Xiang, Shiying Zhang, Irene Shuping Zhao, Xueqi Gan, Yang Zhang

**Affiliations:** ^1^ School of Dentistry Shenzhen University Medical School Shenzhen 518055 China; ^2^ Institute of Oral Science Shenzhen University Shenzhen 518055 China; ^3^ State Key Laboratory of Oral Disease National Clinical Research Center for Oral Diseases West China Hospital of Stomatology Sichuan University Chengdu 610041 China; ^4^ School of Biomedical Engineering Shenzhen University Medical School Shenzhen 518055 China

**Keywords:** exosomes, extracellular vesicles, microenvironment, stimuli, therapeutic efficacy

## Abstract

Extracellular vesicles (EVs) hold significant promise for the prevention and treatment of various diseases. However, the translation of EV‐based therapies into clinical practice faces considerable challenges, particularly in terms of production yield and therapeutic efficacy. Recent studies have emphasized the heterogeneity of EVs and the influence of parental cell microenvironmental signals on their biogenesis, cargo composition, and therapeutic outcomes. This review offers a comprehensive overview of strategies to optimize the therapeutic efficacy of EVs through physical, biochemical, and mechanical modulation. Additionally, it explores how microenvironmental signals affect EV cargoes and the mechanisms by which these signals can improve therapeutic efficacy. The review also addresses current challenges and potential solutions to accelerate the clinical translation of EV therapies. Ultimately, it highlights the potential of microenvironmental modulation in unlocking the full therapeutic capacity of EVs, providing key insights into their production and clinical use for treating various diseases.

## Graphical abstract

Recent studies highlighted the heterogeneity of EVs and the influence of microenvironmental signals on their therapeutic efficacy. This review offers a comprehensive overview of strategies and mechanisms to optimize the therapeutic efficacy of EVs through physical, biochemical, and mechanical modulation. It also proposes current challenges and the potential of microenvironmental modulation in unlocking the full therapeutic capacity of EVs.

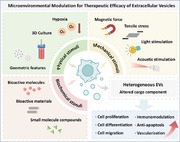

## 1 Introduction

Regenerative medicine focuses on restoring the function of damaged organs or tissues, primarily through the use of stem cells and their secretomes. Recently, extracellular vesicles (EVs) have garnered significant attention as a promising regenerative tool, with a growing body of research investigating their potential to repair and regenerate various organs and tissues.^[^ ^1^ ^]^ EVs are membrane‐bound vesicles secreted by cells, carrying bioactive molecules like proteins, lipids, and nucleic acids.^[^ ^2^ ^]^ Since their contents reflect the characteristics of their parent cells, EVs can replicate certain functions of these cells, influencing numerous physiological processes via intercellular communication.^[^ ^3^ ^]^ For example, they mediate signal transduction by activating cell surface receptors through proteins or bioactive lipid ligands.^[^ ^4^ ^]^ Additionally, EVs can deliver their surface proteins and cytoplasmic contents to target cells by fusing with their plasma membranes or through endocytosis.^[^ ^5^ ^]^ Given their involvement in tissue homeostasis and pathology, EVs have emerged as promising nanosized vehicles for delivering therapeutic agents in regenerative medicine.

From a clinical application perspective, EVs offer potential as off‐the‐shelf therapeutic products, with benefits like lower immunogenicity, improved safety, and favorable regulatory aspects.^[^ ^6^ ^]^ Current applications of EVs in regenerative medicine are diverse and impactful, with evidence showing their ability to promote tissue repair in models of myocardial infarction,^[^ ^7^ ^]^ acute and chronic kidney diseases,^[^ ^8^ ^]^ skeletal muscle repair,^[^ ^9^ ^]^ etc. Their ability to modulate immune responses and promote angiogenesis further enhances their regenerative potential across various tissue conditions.^[^ ^10^ ^]^

However, despite the promise of EV‐based therapies, several challenges remain. One significant issue is the rapid clearance of systemically administered EVs from the bloodstream, limiting their therapeutic efficacy.^[^ ^11^ ^]^ Another challenge in the clinical application of EVs for regenerative medicine is addressing poor yield and low efficacy. Their therapeutic efficacy is heavily dependent on their heterogeneity. Actually, EVs are a highly heterogeneous group of vesicles, differing in size, cargo, surface properties, and origin, which complicates their use but also offers opportunities. The heterogeneous nature of EVs means that their composition and function can be modified to improve therapeutic outcomes. Factors such as the cellular origin, physiological state, and biological compounds play crucial roles in shaping EV heterogeneity, and microenvironmental signals like hypoxia, IFN‐γ conditioning, glucose starvation, or tensile stress can influence EV subtype production and therapeutic effectiveness.^[^ ^12^ ^]^ Adjusting environmental factors or culture conditions to modify EV biogenesis presents a promising strategy for enhancing the therapeutic potential of EV subtypes.

This review aims to provide a comprehensive overview of techniques that have the potential to enhance the therapeutic efficacy of EVs through microenvironmental modulation. It first elaborates on the biogenesis and heterogeneity of EVs and their effects on therapeutic efficacy, then summarizes various environmental signals, including physical, mechanical, and biochemical factors, that serve as key modulators in producing highly therapeutic EVs. Additionally, it explores the underlying mechanisms, current challenges, and potential strategies to address these obstacles, with the goal of encouraging further research into how cellular responses influence the function of EVs. By investigating novel approaches to boost EV therapeutic potency and evaluating their advantages and limitations, this review seeks to advance the field of extracellular vesicles.

## 2 Biogenesis of EVs and Their Classification

EVs generally refer to all lipid bilayer‐enclosed vesicles released by cells that lack functional nuclei. These vesicles encompass various types, including exosomes, microvesicles, endosomes, microparticles, apoptotic bodies, and oncosomes. However, due to the difficulty in distinguishing their subcellular origin, classifying EV subtypes such as exosomes, microvesicles, and endosomes remains challenging. Therefore, in 2024, the International Society for Extracellular Vesicles started an initiative to classify EVs into two primary groups based on their biogenesis: exosomes and ectosomes.^[^ ^13^ ^]^

**Figure 1:**
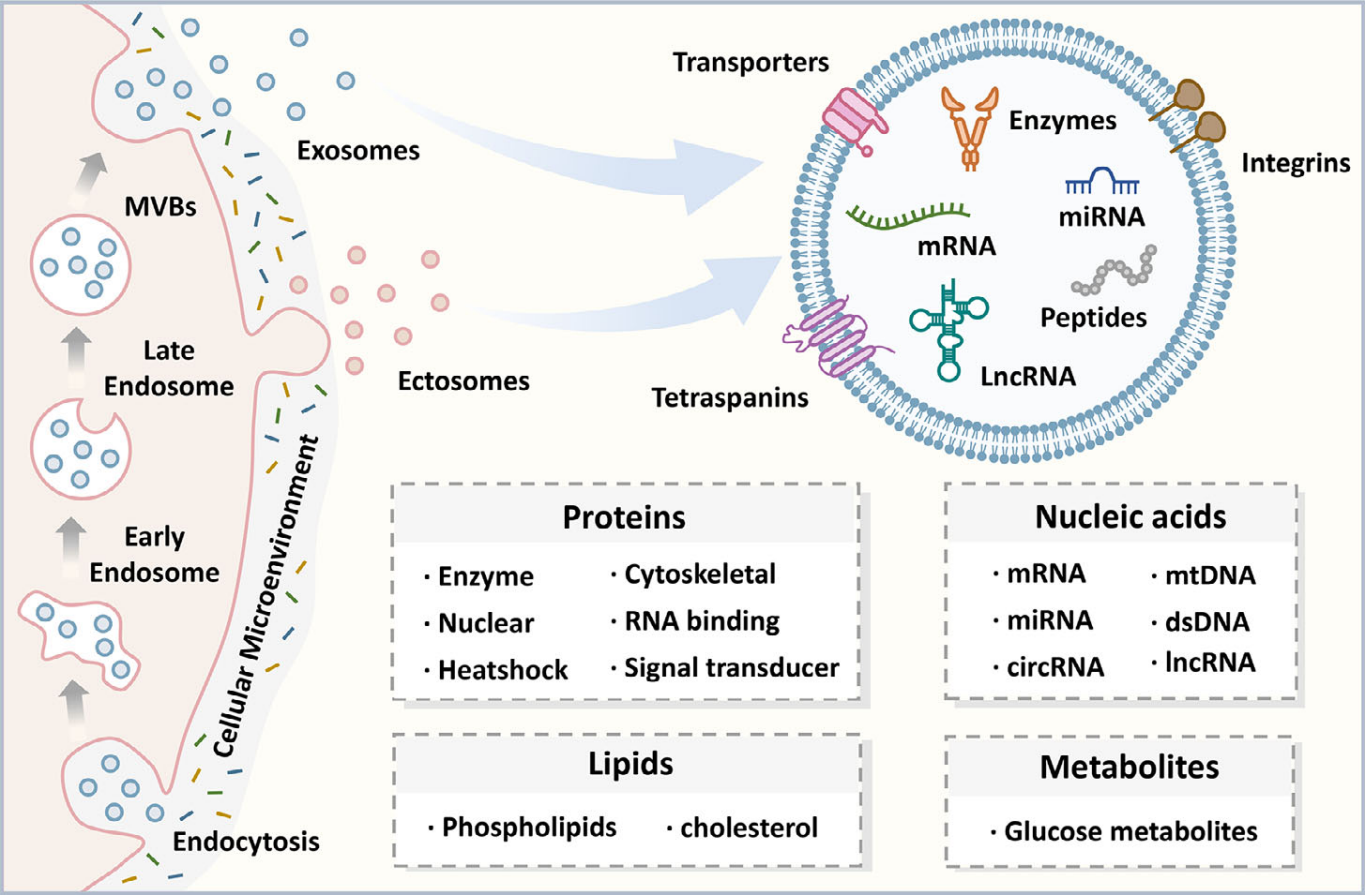
The biogenesis and heterogeneity of Extracellular vesicles. According to the latest recommendation from the International Society for Extracellular Vesicles, EVs are broadly classified into two primary groups based on their biogenesis: exosomes and ectosomes. The distinct processes of their formation, along with variations in cell origin and cellular microenvironment, contribute to the heterogeneity of EVs in EV size and cargo composition.

Exosome biogenesis begins with the invagination of the plasma membrane, leading to the formation of early‐sorting endosomes that contain cell‐surface proteins and extracellular components. These early endosomes mature into late‐sorting endosomes, with the trans‐Golgi network and endoplasmic reticulum playing crucial roles in sorting and defining their contents.^[^ ^5b^ ^]^ Late‐sorting endosomes give rise to multivesicular bodies (MVBs), which house intraluminal vesicles (ILVs), the precursors of exosomes. MVBs can either fuse with lysosomes for degradation or merge with the plasma membrane to release mature exosomes into the extracellular space.^[^ ^14^ ^]^ In contrast, ectosomes are generated through the direct outward budding of the cell membrane. This process involves cytoskeletal rearrangements, membrane protrusion, and selective cargo loading, followed by the scission of the vesicle from the membrane (**Figure** **1**).

The cargoes of EVs primarily consist of proteins, nucleic acids, lipids, metabolites, and carbohydrates (Figure 1).^[^ ^15^ ^]^ They are loaded by different sorting mechanisms, including Endosomal Sorting Complex Required for Transport (ESCRT)‐dependent and ESCRT‐independent pathways, which can be regulated by environmental stress factors such as hypoxia.^[^ ^16^ ^]^ To be specific, the protein content of EVs predominantly originates from the cytosol, plasma membrane, or endocytic membranes of the parent cell.^[^ ^15^ ^]^ They reflect their cellular origin and vary depending on the host cell type. In contrast, miRNAs in EVs typically originate from the cytoplasm, nucleus, or specific organelles and their content can be influenced by specific physiological and pathological conditions of parent cells.^[^ ^16^ ^]^ Under certain scenarios, miRNAs might be more abundant in EVs than in their parent cells, especially within the small RNA population.^[^ ^17^ ^]^

## 3 The Heterogeneity of EVs and Therapeutic Efficacy

Since the first report of EVs in 1967, it has been gradually recognized that EVs represent a heterogeneous population of vesicles with distinct sizes, cargoes, surface characteristics, and functions.^[^ ^18^ ^]^ This heterogeneity of EVs mainly comes from the cell origin (inter‐heterogeneity) and microenvironmental conditions (intra‐heterogeneity) as Randy Carney recently suggested.^[^ ^19^ ^]^ Both two factors affect the biological pathways of EVs, which subsequently lead to alterations in the size, and cargo composition of EVs that eventually determine their therapeutic efficacy.^[^ ^18a^ ^]^  For inter‐heterogeneity, EVs from different cell types offer unique therapeutic potential for specific clinical needs (**Tables** **1**, **2**, **3**). To be specific, macrophage‐derived exosomes primarily modulate immune responses, making them useful in immunotherapy and the treatment of inflammatory diseases.^[^ ^20^ ^]^ In contrast, stem cells‐derived EVs predominantly display pro‐angiogenic and anti‐inflammatory properties that support tissue repair and regeneration.^[^ ^14^, ^21^ ^]^ Endothelial cells‐derived EVs demonstrate strong pro‐angiogenic properties and endothelial protection, making them ideal for supporting vascular remodeling.^[^ ^22^ ^]^ Even within the same cell type, the subtype from different tissues significantly influences the EV production, protein composition, miRNA profile, and functional properties.^[^ ^23^ ^]^ For instance, EVs derived from bone marrow mesenchymal stem cells (BMSCs) exhibit significant advantages in immunoregulation, whereas EVs from adipose‐derived stem cells (ADSCs) demonstrate superior pro‐angiogenic effects in comparison to those from BMSCs.^[^ ^21^, ^23^ ^]^

**Table 1:** Physical modulation for optimizing the regenerative effects of EVs.

Physical signals	Disease treatment	Parental cells	Altered cargos	Main findings	Refs.
Hypoxia	myocardial infarction	BMSCs	miR‐210	increased miR‐210 in EVs promoted angiogenesis, survival, and migration of cardiomyocytes	[26]
	myocardial infarction	BMSCs	miR‐125b‐5p	increased miR‐125b‐5p in EVs inhibited cardiomyocyte apoptosis	[40a]
	myocardial infarction	BMSCs	miRNA‐24	increased microRNA‐24 in EVs inhibited cardiomyocyte apoptosis	[40b]
	myocardial infarction	MSCs	LncRNA‐UCA1	increased LncRNA‐UCA1 in EVs inhibited cardiomyocyte apoptosis	[40c]
	osteoarthritis	BMSCs	miR181c‐5p, miR‐18a‐3p, miR‐376a‐5p, miR‐337‐5p	altered miRNAs in EVs promoted proliferation and migration and suppressed apoptosis of chondrocyte	[21a]
	bone fracture	UCMSCs	miR‐126	increased miR‐126 in EVs promoted proliferation, migration, and tube formation of UVECs	[12b]
	osteoarthritis	BMSCs	miR‐216a‐5p	increased miR‐216a‐5p in EVs promoted proliferation and migration and suppressed apoptosis of chondrocyte	[25]
	wound healing	ADSCs	miR‐21‐3p, miR‐126‐5p, miR‐31‐5p, miR‐99b and miR‐146‐a	increased miR‐21‐3p, miR‐126‐5p, miR‐31‐5p and decreased miR‐99b and miR‐146‐a in EVs promoted fibroblast proliferation and migration	[21b]
	wound healing	ADSCs	circ‐Snhg11	increased circ‐Snhg11 in EVs promoted endothelial cell proliferation, invasion, and tube formation	[41c]
	wound healing	AMSCs	KGF, ECF, FGF1, TGF‐β, VEGF	increased KGF, ECF, FGF1, TGF‐β, and VEGF in EVs facilitated the proliferation of keratinocytes and optimized the properties of fibroblasts.	[41d]
	spinal cord injury	BMSCs	miR‐216a‐5p	increased miR‐216a‐5p in EVs regulated microglia M1/M2 polarization	[42]
	ischemic diseases	ADSCs	VEGF	increased VEGF in EVs promoted angiogenesis of UVECs	[41a]
	ischemic diseases	OMMSCs	miR‐612	increased miR‐612 in EVs promoted angiogenesis of BMECs	[41b]
Hydrogel	wound healing	ADSCs	–	enhanced expression of basal and suprabasal cytokeratins of keratinocytes	[37]
Microcarriers	Parkinson's disease	SHEDs	–	suppressed 6‐OHDA‐induced apoptosis of dopaminergic neurons	[24]
Graphene scaffold	Alzheimer's disease	UCMSC	neprilysin, insulin‐degrading enzyme, heat shock protein 70	Altered 195 kinds of miRNAs and proteins in EVs reduced secreted and intracellular Aβ of human neuroblastoma cells	[34a]
Hydroxyapatite	–	BMSCs	HMGB1	increased HMGB1 in EVs improved hUVEC cell proliferation, migration, tube formation	[34b]
GelMA hydrogel	spinal Cord Injury	MSCs	SOX2	exerted a stronger anti‐inflammatory effect and exhibited the attenuation of glial/ fibrotic scar formation‐induced	[35b]
Collagen hydrogel	bone defect	PDLSCs	Runx2, OPN, OCN, COL1A1, ALP	enhanced proliferation and migration and inhibited apoptosis of BMSCs	[36]
Spheroid	hair loss	DPCs	miR‐218‐5p	increased miR‐218‐5p in EVs‐regulated hair follicle growth	[32]
	skin aging	HDFs	miR‐196a, miR‐133a, miR‐223,	induced collagen synthesis and antiaging	[33]
	–	MSCs	miR‐210	increased miR‐210 in EVs improved angiogenesis and neurogenesis of UVECs	[35a]
Bioreactor	acute kidney injury	UVECs	–	Improved renal function, attenuated pathological changes of renal tubules, reduced inflammatory factors, and repressed T cell and macrophage infiltration.	[38b]
	–	BMSCs	miR‐21, miR‐22, cytokines	higher miR‐21, miR‐22, and altered proteins in EVs enhanced immunomodulatory potentials	[39]
Stiffness	–	MSCs	proteins and lipids	stiff‐EVs induced a higher phagocytic efficiency of macrophages	[47]
Nanotopography	bone defect	BMSCs	RAB27B, SMPD3	promoted osseointegration of titanium implants	[43b]
	aged skeletal muscle injuries	MPCs	–	promoted the myogenic differentiation of young MPCs.	[44b]
	–	BMSCs	TGFβ, AMPK, FoxO	meditated osteogenesis of BMSCs through miRNA	[44a]
Pore shape and size	bone defect	MC3T3 cells	–	EVs derived from the triangle pore scaffolds promoted BMSCs osteogenic differentiation when compared to EVs derived from square scaffold designs	[45]
Pore size	–	myeloid cells	FIZZ1, CD206, ARG‐1, CD86, TNFα	affected the transcriptome and viability of CD3+ T cells	[46]

Except for the inter‐heterogeneity, heterogeneity exists even among EVs from the same parental cells when cultured under different microenvironments.^[^ ^18a^ ^]^ These factors include but are not limited to pH, cellular stress, mechanical stimuli, starvation, hypoxia, inflammation, and hyperglycemia. For example, only EVs derived from stem cells cultured in a 3D bioreactor under oxidative stress conditions demonstrated the ability to protect neurons from apoptosis compared to EVs from standard 2D cultures.^[^ ^24^ ^]^ Similarly, EVs derived from stem cells under hypoxic conditions generally exhibit improved therapeutic potential, enhanced cargo delivery, increased regenerative capacity, and better cell proliferation support compared to EVs from cells cultured under normal conditions.^[^ ^12^, ^25^ ^]^

The heterogeneous characteristics of EVs pose challenges to the clinical translation of EV‐based therapies, as it complicates the standardization process. Varying size and cargo composition of EVs can lead to inconsistent therapeutic outcomes, making it difficult to achieve predictable and reproducible effects in patients. Despite these challenges, EV heterogeneity also presents potential advantages. By isolating and characterizing specific subpopulations, researchers can select EVs with optimal properties for targeted therapies. To be specific, proteins, lipids, RNA (mRNA, miRNA, lncRNA), and even metabolites are selectively packaged during EV biogenesis, practically during the formation of multivesicular bodies and their fusion with the plasma membrane.^[^ ^16^ ^]^ This selective packing can influence the surface proteins that determine their ability to interact with specific recipient cells and cargo composition, which greatly influences the therapeutic effects of EVs. For instance, cellular pathways involved in EV biogenesis can be modified under hypoxia to prioritize the packaging of miRNA‐210, thereby enhancing their cardiac regeneration.^[^ ^26^ ^]^ Besides, it is reported that exosomes are typically derived from intracellular endosomal compartments and may carry more specialized cargo (e.g., RNA or proteins related to cell signaling).^[^ ^27^ ^]^ Ectosomes, on the other hand, bud directly from the plasma membrane and express CD47 which helps evade immune detection, thereby extending their circulation time in the body and enhancing their ability to target multiple sites simultaneously.^[^ ^27^ ^]^ Besides the cargo composition, different EV subpopulations can express varying levels and types of surface proteins (e.g., tetraspanins like CD9, CD63, CD81, or integrin).^[^ ^28^ ^]^ These surface markers influence how EVs interact with recipient cells because EVs with specific surface proteins may preferentially bind to particular cell types or tissues. For example, EVs from macrophages might naturally target immune cells to modulate the immune response,^[^ ^20b^ ^]^ while EVs from Schwann cells could promote neurite outgrowth and aid in nerve regeneration.^[^ ^29^ ^]^ Therefore, modulating the biogenesis pathways to incorporate specific targeting ligands or modifying surface proteins can enhance the targeting capabilities of EVs for therapeutic purposes. Moreover, the biogenesis and environmental signals affect the size of EVs, ranging from 30 to 1000 nm.^[^ ^13^ ^]^ The heterogeneous size of these EVs influences their ability to penetrate tissues, interact with cells, cross biological barriers (e.g., the blood‐brain barrier), and eventually the therapeutic efficacy.^[^ ^30^ ^]^ To sum up, the heterogeneity of EVs is fundamental to their therapeutic efficacy because it dictates the EV cargo, surface markers, size, etc. By understanding and manipulating the cellular processes involved in EV biogenesis and heterogeneity, researchers can design EVs that are optimized for specific therapeutic applications.

## 4 Microenvironmental Modulation of EVs for Disease Treatment

**Figure 2:**
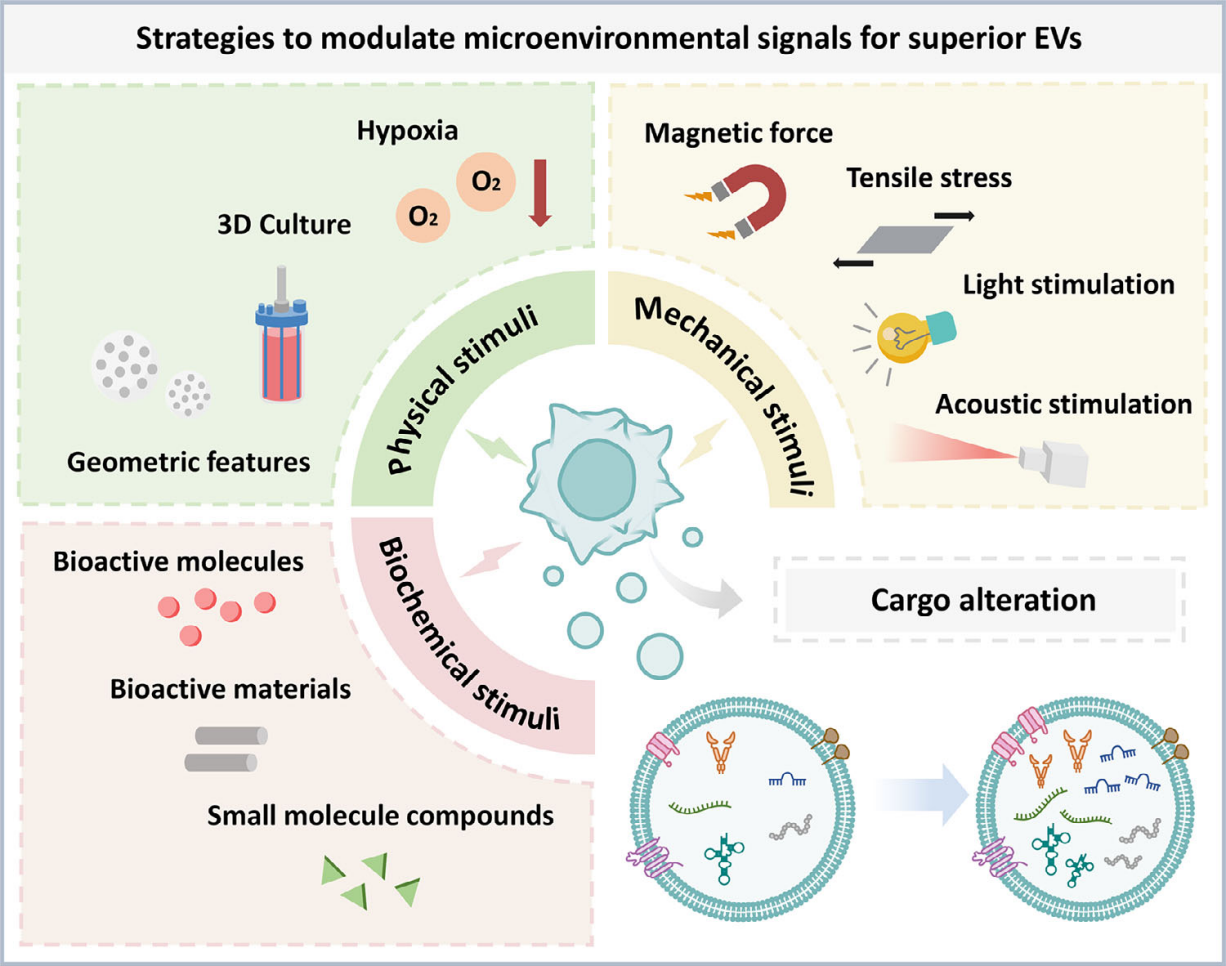
Microenvironmental modulation for therapeutic efficacy of extracellular vesicles. The therapeutic efficacy of EVs can be regulated through physical, biochemical, and mechanical approaches. These microenvironmental signals include but are not limited to 3D culture, hypoxia, geometric features, magnetic forces, tensile stress, blue light, acoustic stimulation, bioactive molecules, small molecule compounds, and biomaterials.

The production of EVs can be influenced by the cell origin and environmental conditions of parent cells. By inducing specific types of EVs with desired characteristics, their therapeutic potential can be spontaneously enhanced for specific therapeutic applications. Among these factors, controlling the culture environment, particularly the microenvironmental signals, offers the most convenient, cost‐effective, and efficient way to regulate EV function (Table 1, 2, 3). Based on existing literature, these microenvironmental signals primarily encompass physical, mechanical, and biochemical factors (**Figure** **2**).

**Table 2:** Mechanical modulation for optimizing the regenerative effects of EVs.

Mechanical signals	Disease treatment	Parental cells	Altered cargo profile	Main findings	Refs.
Magnetic force	bone defect	BMSCs	miR‐1260a	increased miR‐1260a in EVs enhanced osteogenesis of BMSCs and angiogenesis of UVECs	[53a]
	peripheral nerve injury	schwann cells	miR‐23b‐3p	increased miR‐23b‐3p in EVs enhanced neurite outgrowth and nerve regeneration	[29]
	peripheral nerve injury	schwann cells	C5aR1	increased C5aR1 in EVs promoted axon growth, angiogenesis, and inflammatory regulation	[54]
	wound healing	MSCs	–	enhanced cell proliferation, migration, and angiogenesis of UVECs	[53b]
	wound healing	BMSCs	miR‐21‐5p	increased miR‐21‐5p in EVs enhanced cell proliferation, migration, and angiogenesis of UVECs	[53c]
Shock wave	myocardial infarction	UVECs	miR‐19a‐3p	increased miR‐19a‐3p in EVs enhanced endothelial tube formation and proliferation	[56]
Tension	bone defect	PDLCs	–	inhibited IL‐1β Production and pyroptosis in LPS‐primed macrophages	[49a]
	bone defect	PDLCs	miR‐21‐5p	increased miR‐21‐5p in EVs improved the proliferation of MC3T3‐E1 cells and enhanced the osteogenic differentiation of osteoblasts	[49b]
	bone defect	BMECs	FGF‐1, TGF‐β1	improved angiogenesis and osteogenesis of BMECs	[12e]
	Hypertension	VSMCs	Caveolin‐1	increased Caveolin‐1 in EVs increased the mineralization potential of VSMCs	[50]
Ultrasound	acute kidney injury	BMSCs	–	increased proliferation, and reduced inflammation and apoptosis in the kidney	[55]
Blue light	cutaneous burn	UVECs	miR‐135b‐5p, miR‐499a‐3p	increased miR‐135b‐5p, miR‐499a‐3p in EVs enhanced UVECs proliferation	[57]

**Table 3:** Biochemical modulation for optimizing the regenerative effects of EVs.

Biochemical signals	Disease treatment	Parental cells	Altered cargo profile	Main findings	Refs.
LPS	wound healing	UCMSCs	let‐7b	increased let‐7b in EVs converted inflammatory THP‐1 cells to M2 polarization	[60]
	myocardial infarction	BMSCs	TNFα, IL‐6 and IL‐1β	promoted M2 macrophage polarization	[61]
IL‐1β	sepsis	UCMSCs	miR‐146a	increased miR‐146a in EVs promoted M2 macrophage polarization	[62a]
	brain inflammation	BMSCs	BDNF, IL‐1Ra, VEGF, IL‐10, NGF	inhibited LPS‐induced inflammatory responses in astrocytes	[62b]
	osteoarthritis	ADSCs	miR‐155‐5p, miR‐146a‐5p, miR196b‐5p, miR‐125a‐3p, miR‐134‐5p, miR‐520c‐3p	increased chondro‐protective miRNAs in EVs and penetrated cartilage fast	[62c]
TNFα	bone defect	ADSCs	Wnt‐3a	increased Wnt‐3a in EVs promoted the proliferation and osteogenic differentiation of osteoblastic cells	[63a]
	bone defect	GMSCs	miR‐1260b	increased miR‐1260b in EVs enhanced M2 macrophage polarization and inhibited periodontal bone loss	[65]
	urethral stricture	UCMSCs	miR‐146a	increased miR‐146a in EVs mediated myofibroblast differentiation and the release of proinflammatory factors	[63b]
	acute liver failure	UCMSCs	miR‐299‐3p	increased miR‐299‐3p in EVs affected the release of proinflammatory cytokines of macrophages	[64]
IFNγ	osteoarthritis	ADSCs	miR‐210, miR245	was chondro‐protective and M2 macrophage polarizing	[59a]
IFNγ & TNFα	–	ADSCs	miR‐34, miR‐146	increased miRNAs in EVs promoted M2 macrophage polarization	[59b]
TGF‐β1	osteoarthritis	BMSCs	miR‐135b	increased miR‐135b in EVs promoted M2 macrophage polarization	[59c]
TGF‐β1, IFNγ	graft vs host disease	UCMSCs	IDO	increased IDO in EVs promoted the transformation of mononuclear cells to Treg cells	[59d]
BMP2	bone defect	macrophages	–	activated autophagy during osteogenic differentiation of BMSCs	[66b]
Erythropoietin	chronic kidney disease	BMSCs	miR‐299, miR‐499, miR‐302, miR‐200	changed miRNA in EPO‐MVs enhanced protective effects following renal injury	[66a]
Melatonin	wound healing	BMSCs	–	promoted M2 macrophage polarization	[69]
	chronic kidney disease	ADSCs	miR‐4516	increased miR‐4516 in EVs improved functional recovery and vessel repair of MSCs with chronic kidney disease	[68]
Atorvastatin	wound healing	BMSCs	miR‐221‐3p	increased miR‐221‐3p in EVs promoted the proliferation, migration, tube formation, and VEGF level of endothelial cells	[73]
Curcumin	osteoarthritis	BMSCs	miR‐143, miR‐124	increased miR‐143 and miR‐124 in EVs suppressed chondrocyte apoptosis	[72a]
DMOG	bone defect	BMSCs	–	stimulated angiogenesis in UVECs	[71]
Kartogenin	osteoarthritis	BMSCs	COL2, Prg4, Agg, SOX‐9	promoted chondrocyte migration and proliferation	[72b]
Lithium chloride	focal cerebral ischemia	BMSCs	miR‐1906	increased miR‐1906 in EVs provided profound neuroprotection and neurological recovery	[74]
BCP ceramics	Bone defect	macrophages	15 miRNAs	promoted M2 macrophage polarization, enhanced angiogenic differentiation of UVECs, and induced osteogenesis of MSCs	[20b]
Silicate ceramics	bone defect	BMSCs	miR‐146a	increased miR‐146a in EVs promoted angiogenesis of UVECs	[75a]
	myocardial infarction	EPCs	miR‐126a‐3p	increased miR‐126a‐3p in EVs promotes angiogenesis of UVECs	[75b]
Nitric oxide releasing polymer	ischemic damage	UCMSCs	miR‐126, VEGF	increased VEGF and miR‐126 levels in EVs promoted angiogenesis of UVECs	[75c]

Abbreviation: DMOG, dimethyloxaloylglycine; BCP, biphasic calcium phosphate; BMSCs, bone‐marrow derived mesenchymal stem cells; ADSCs, adipose‐derived stem cells; UCMSC, umbilical cord mesenchymal stem cells; SHEDs, stem cells from human exfoliated deciduous teeth; PDLSCs, periodontal ligament stem cells; DPCs, dermal papilla cells; HDFs, human dermal fibroblasts; UVECs, umbilical vein endothelial cells; BMECs, brain microvascular endothelial cells; GMSCs, gingival mesenchymal stem cells; iPSCs, induced pluripotent stem cells; RGD, Arginine‐Glycine‐Aspartic acid peptide, AMSCs, amniotic membrane stem cells; EPCs, endothelial progenitor cells; MPCs, muscle stem/progenitor cells; VSMCs, vascular smooth muscle cells.

### 4.1 Physical Factors to Modulate EVs for Improved Therapeutic Efficacy

#### 4.1.1 3D Culture

**Figure 3:**
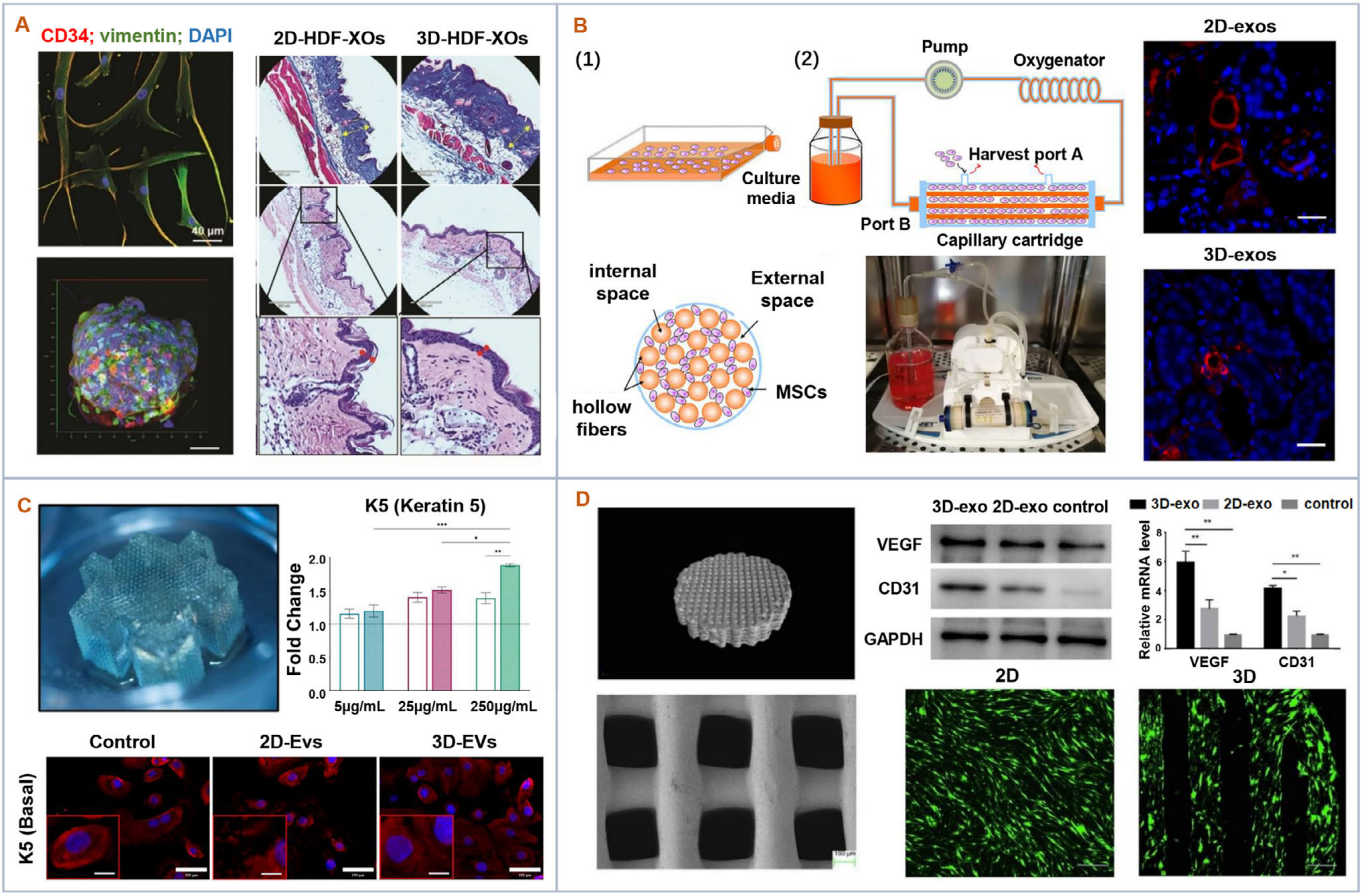
3D culture to enhance therapeutic efficacy of extracellular vesicles. A) Spheroids were generated by transferring human dermal fibroblasts into ultralow attachment flasks. The 3D HDF‐EVs demonstrated greater improvement in repairing UV‐induced collagen fiber damage compared to 2D HDF‐EVs. Adapted with permission.^[^ ^33^ ^]^ Copyright 2019, ACS Nano. B) A hydroxyapatite scaffold was prepared by 3D printing. The 3D‐EVs‐treated groups showed significantly higher VEGF and CD31 expression, indicating a superior angiogenesis effect over the 2D group. Adapted with permission.^[^ ^34b^ ^]^ Copyright 2021, Stem Cell Research. C) 3D hydrogels with a distinctive macro‐ and microarchitecture were printed. 3D‐EVs enhanced the expression of basal cytokeratin. Adapted with permission.^[^ ^37^ ^]^ Copyright 2023, Tissue Engineering and Regenerative Medicine. D) 3D‐EVs were produced within the hollow fiber bioreactor‐based 3D culture system, which enhanced the renoprotective efficacy in cisplatin‐treated mice. Adapted with permission.^[^ ^38b^ ^]^ Copyright 2020, Stem Cell Research & Therapy.

Cells interact with their surroundings which more closely mimics the in vivo conditions in 3D culture (**Figure** **3**).^[^ ^31^ ^]^ This advantage can be utilized to produce EVs that closely resemble their parental cells in terms of cargo profile, lipid composition, and protein level. For example, the typical spheroid culture method significantly enhanced the efficacy of EVs derived from dermal papilla cells and dermal fibroblasts by increasing the content of therapeutic factors such as miR‐218‐5p, miR‐196a, miR‐133a, and miR‐223, further improving their potential in hair regrowth^[^ ^32^ ^]^ and antiaging treatments.^[^ ^33^ ^]^ Besides, with the aid of 3D scaffolds, EVs derived from stem cells exhibited significant alterations in their cargo compared to 2D tissue culture plates.^[^ ^34^ ^]^ These cargo changes enhanced angiogenic, osteogenic differentiation, and immunomodulatory capabilities, ultimately boosting the regenerative potential of stem cell‐derived EVs and improving neuroprotective,^[^ ^24^, ^34^, ^35^ ^]^ pro‐osteogenic,^[^ ^36^ ^]^ and epithelialization functions.^[^ ^36^, ^37^ ^]^

As another type of 3D culture, bioreactor platforms are commonly used for the 3D culture of parental cells due to their superior control over environmental parameters such as temperature, pH, oxygen, and carbon dioxide. They are reported to alter the cargo content of EVs from BMSCs (bone‐derived mesenchymal stem cells) and endothelial cells to upregulate the metabolic, autophagy, and ROS‐related proteins compared to traditional 2D cultures for the treatment of neurological disorders and acute kidney injury.^[^ ^38^ ^]^ This effect was also found in 3D aggregates of BMSCs cultured under wave motion bioreactor, which significantly enhanced the immunomodulatory effects of produced EVs through higher miR‐21 and miR‐22 expression compared to 2D cultures.^[^ ^39^ ^]^

While 3D culture systems offer several advantages over traditional 2D methods, they also present challenges, particularly in retrieving EVs from dense, non‐porous scaffolds like hydrogels. In these 3D environments, EVs can become trapped within the matrix, often requiring additional steps such as enzymatic digestion to release them. However, these processes may compromise the integrity of EVs and bioactivity. Moreover, the reproducibility and scalability of 3D constructs remain critical challenges, heavily influenced by the chosen manufacturing techniques. Future research should prioritize the development of thermosensitive or enzymatically degradable materials that can degrade under controlled conditions, facilitating the release of EVs into the culture medium without harming their function.

#### 4.1.2 Hypoxia

**Figure 4:**
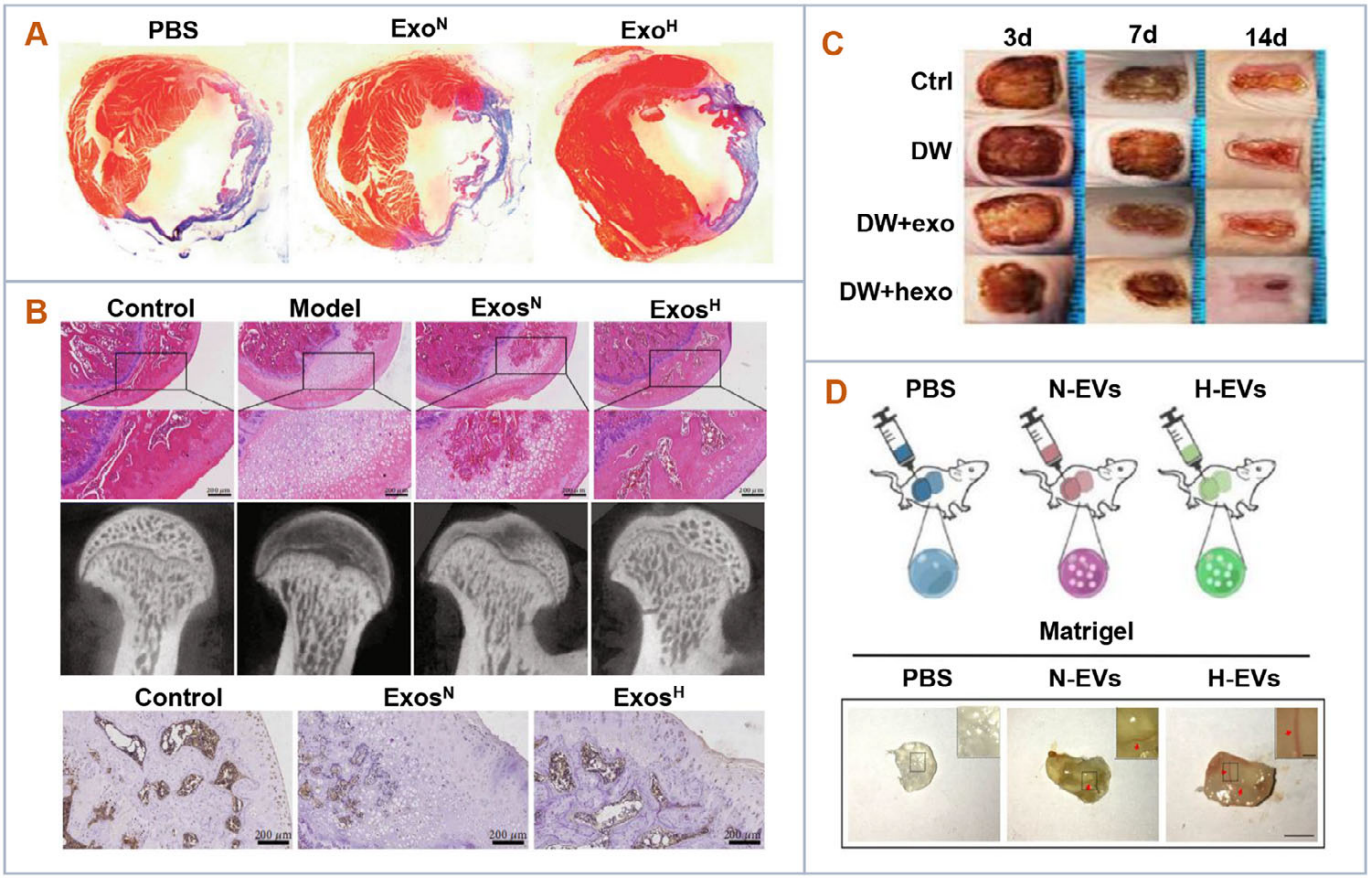
Hypoxia conditions to enhance the therapeutic efficacy of extracellular vesicles. A) Hypo‐EVs enhanced cardiac function and reduced fibrosis after myocardial infarction. Adapted with permission.^[^ ^26^ ^]^ Copyright 2018, Artificial Cells Nanomedicine and Biotechnology. B) Hypo‐EVs protected bone tissue and stimulated angiogenesis in a rat osteonecrosis model.^[^ ^39^ ^]^ Copyright 2021, BioMed Research International. C) Hypo‐EVs accelerated diabetic wound healing. Adapted with permission.^[^ ^21b^ ^]^ Copyright 2021, Journal of Nanobiotechnology. D) Hypo‐EVs significantly promoted blood vessel formation in the Matrigel plug assay. Adapted with permission.^[^ ^41b^ ^]^ Copyright 2021, Journal of Nanobiotechnology.

Physiological oxygen levels typically range between 2% and 9%, whereas standard laboratory conditions expose cells to ambient oxygen levels of ≈21%.^[^ ^23b^ ^]^ This discrepancy has prompted numerous researchers to explore the use of hypoxic conditions to more accurately simulate in vivo biological processes. Under hypoxia, cells undergo a series of adaptive changes regulated by hypoxia‐inducible factors (HIFs). These adaptive changes not only enhance the survival and function of MSCs but also improve the therapeutic efficacy of EVs derived from these cells (**Figure** **4**). For instance, hypoxia‐conditioned MSC‐derived EVs (hypo‐EVs) have been shown to enhance cardiac repair and protect against myocardial infarction by upregulating specific miRNAs and lncRNAs, such as miR‐210 and lncRNA‐UCA1.^[^ ^26^, ^40^ ^]^ These hypo‐EVs from MSCs displayed enhanced angiogenic capacity and superior therapeutic effects for ischemic and traumatic conditions through the upregulation of proteins and microRNAs derived from different sources of EVs.^[^ ^12^, ^21^, ^25^, ^41^ ^]^ Except for affecting angiogenesis, the Hypo‐EVs from BMSCs also facilitate neurological recovery following spinal cord injury through the polarization of microglia.^[^ ^42^ ^]^ Although oxygen concentrations between 0.5% and 5% are also utilized in some studies, the most employed oxygen concentration for hypoxic conditions is 1%. To date, no studies have investigated the impact of varying oxygen levels on EV efficacy, and thus, the optimal conditions for maximizing their therapeutic potential remain undetermined.

#### 4.1.3 Geometric Features

Geometric features refer to the measurable attributes and spatial properties of scaffolds used in tissue engineering, including topography, porosity, stiffness, and surface roughness. These features not only influence cellular behavior but also significantly impact the efficacy of EVs.^[^ ^43^ ^]^ For example, the nanotopography on titanium surfaces was reported to enhance osteogenesis and myogenic differentiation by upregulating gene expression and miRNA content in muscle stem cells and BMSC‐derived EVs for the treatment of bone defects and aged skeletal injuries.^[^ ^43^, ^44^ ^]^ Except for topography, Pore size and shape affect the therapeutic efficacy of EVs as well. For instance, EVs derived from ADSCs in titanium implants with triangular pore structures and larger pore sizes promoted BMSCs osteogenic differentiation compared to scaffolds with square pore structures and smaller pore sizes.^[^ ^45^ ^]^ Likewise, polymethyl methacrylate scaffolds with 40 µm spherical pores augmented the effect of myeloid cell‐derived EVs on T cells through a pore‐size‐dependent mechanism.^[^ ^46^ ^]^

In addition to these geometric factors, extracellular matrix (ECM) stiffness plays a critical role in regulating cargo sorting into MSC‐EVs.^[^ ^47^ ^]^ This modulation of key proteins and lipids, such as vesicular transport‐related proteins and autophagy‐related lipids, directly affects the secretion and target behavior of these EVs.^[^ ^47^ ^]^ Beyond geometric and ECM‐related features, other physical properties, such as conformation, viscoelasticity, and degradation behavior of the ECM, also contribute to the EV heterogenicity and therapeutic efficacy, which remains less explored yet.

### 4.2 Mechanical Stimuli to Modulate EVs for Improved Therapeutic Efficacy

#### 4.2.1 Tensile stress

**Figure 5:**
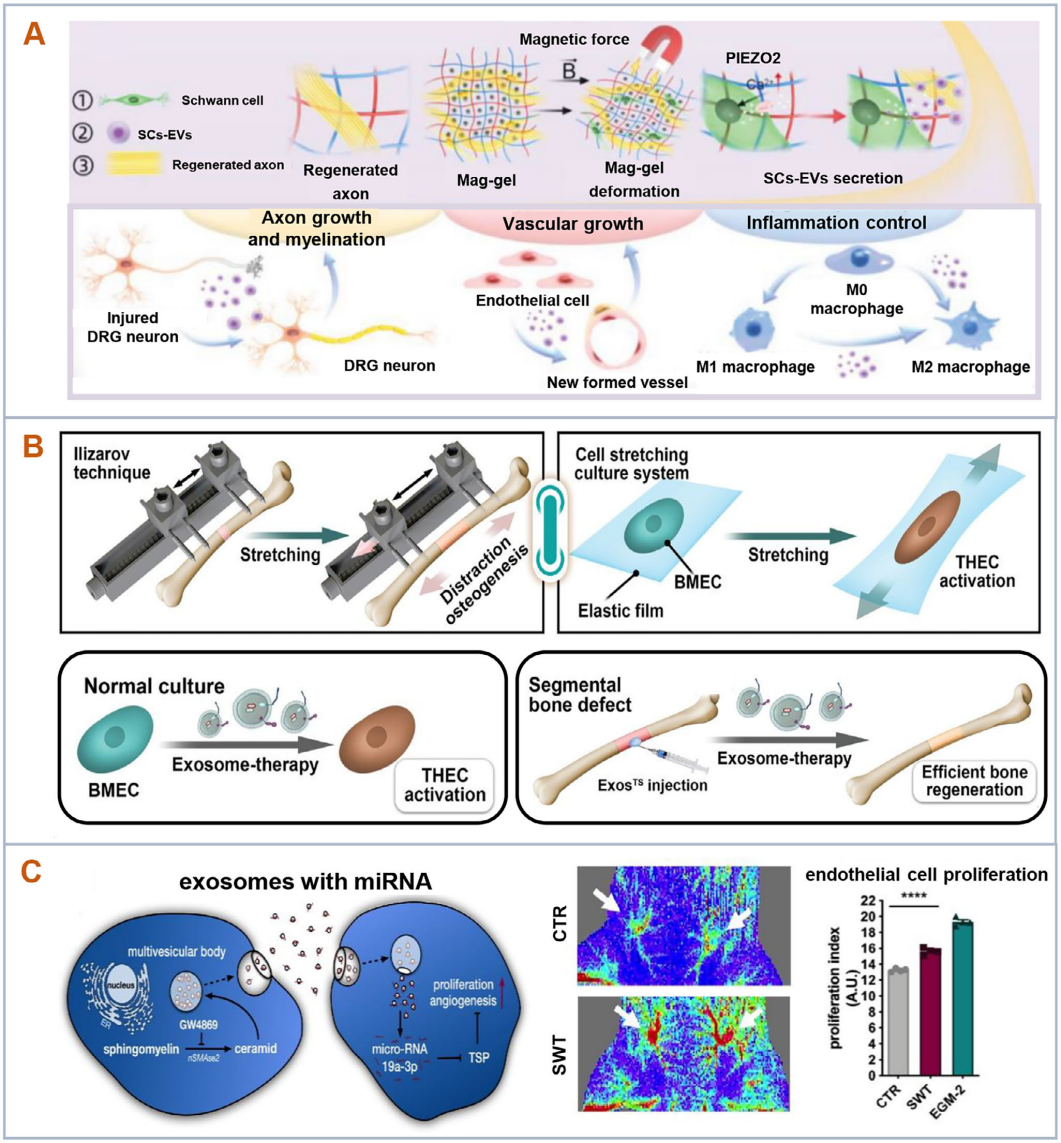
Mechanical factors to enhance the therapeutic efficacy of extracellular vesicles. A) A novel magnetic nerve scaffold was designed to enhance in situ production of EVs for improved peripheral nerve repair, newly formed vessels, and inflammation control. Adapted with permission.^[^ ^55^ ^]^ Copyright 2024, Advanced materials. B) The Ilizarov technique was utilized to facilitate the distraction osteogenesis process. The tension stress during this process enhanced the osteogenesis effects of EVs. Adapted with permission.^[^ ^12e^ ^]^ Copyright 2024, Advanced Science. C) Shock wave therapy (SWT) served as a physical stimulus that triggered the release of EVs, which subsequently promoted the angiogenesis of tissue‐engineered grafts. Adapted with permission.^[^ ^57^ ^]^ Copyright 2020, Cardiovascular Research.

Mechanosensing plays a critical role in mediating communication between cells and their surrounding microenvironment. It refers to the cellular response to mechanical forces, which can trigger the release of EVs that facilitate communication both locally and systemically.^[^ ^48^ ^]^ Tension is one such mechanical stimulus that cells can detect in various tissues, including muscles, bones, and blood vessels, potentially influencing both cellular behavior and the composition of EVs (**Figure** **5**). For example, EVs secreted by periodontal ligament stem cells under tensile stress inhibited IL‐1β production and thereby promoted periodontal regeneration.^[^ ^49^ ^]^ Similarly, EVs from endothelial cells and vascular smooth muscle cells under 10% mechanical stretch (0.5 Hz) promoted osteogenesis by enhanced TGF‐β1 and caveolin‐1 content.^[^ ^12^, ^50^ ^]^ To be noted, no all EVs upon mechanical stimuli are beneficial for the disease. For example, EVs from MC3T3‐E1 cells subjected to 20% cyclic tension (1 Hz) exacerbated bone resorption in peri‐implantitis by increased circ_0008542 content.^[^ ^51^ ^]^ However, this enlightens us to control the tension stimulus to reduce the circ_0008542 expression in EVs to effectively reverse bone loss in implants.^[^ ^51^ ^]^ Collectively, these pioneering studies demonstrated that mechanical stimuli are an importance factor of microenvironment cues to modulate the therapeutic efficacy of EVs. However, we have to keep rational that most studies in this area focused on a single cell type, despite tissue regeneration involving the coordinated activity of multiple cell types. For instance, both osteoclasts and osteoblasts are involved in bone formation and may respond differently to tension. Therefore, it is crucial to investigate how different cell types interact with one another through EVs under tensile stress and how these interactions influence subsequent tissue regeneration.

#### 4.2.2 Magnetic Force

Magnetic force refers to the force experienced by a charged particle or a current‐carrying conductor when placed in a magnetic field. This force can alter the direction of particle motion and convert electrical energy into mechanical work. Recent studies have highlighted the significant potential of magnetic forces in enhancing the therapeutic efficacy of EVs for regenerative medicine.^[^ ^52^ ^]^ Cells exposed to magnetic forces exhibit increased activity, leading to enhanced EV biogenesis and improved therapeutic outcomes.^[^ ^52^ ^]^ miRNAs are critical functional components of EVs in response to magnetic forces, playing a central role in cellular communication and the regulation of biological processes.^[^ ^52^ ^]^ For instance, magnetic stimulation enhanced MSC‐EV‐mediated intercellular communication, promoting osteogenesis and angiogenesis through the upregulation of miR‐1260a and miR‐21‐5p.^[^ ^53^ ^]^ Besides, EVs derived from Schwann cells under magnetic force exhibit unique neurite outgrowth and promote nerve regeneration by transferring miR‐23b‐3p and C5aR1.^[^ ^29^, ^54^ ^]^ To be noted, one study used in situ magnetic field stimulation to enhance the local production of EVs for peripheral nerve repair. In this case, it is possible that the magnetic field itself may have a direct therapeutic effect on peripheral nerves, in addition to the promoting effects by EVs.^[^ ^54^ ^]^

Importantly, magnetic nanoparticles have already received approval from the U.S. Food and Drug Administration (FDA) for clinical use, underscoring their safety for therapeutic applications. However, several challenges remain, not limited to the potential contamination of EVs with magnetic nanoparticles during isolation, the need to determine optimal concentrations and power frequencies for treatment, and the lack of precise understanding regarding the mechanisms of action.

#### 4.2.3 Light and Acoustic Stimulation

A promising alternative to mechanical stimulation for enhancing the efficacy of EVs is the use of light and sound waves, offering a more cost‐effective approach. High‐intensity focused ultrasound (HIFU), applied at a customized central frequency, has been shown to upregulate proliferative and regenerative pathways while simultaneously suppressing inflammation, particularly in the treatment of acute kidney injury.^[^ ^55^ ^]^ Similarly, shock wave therapy (SWT) has been found to induce the release of angiogenic EVs containing miR‐19a‐3p, which improved vascularization, reduced myocardial fibrosis, and enhanced cardiac function following myocardial ischemia.^[^ ^56^ ^]^ Furthermore, blue light at 455 nm illumination significantly enhanced the proangiogenic potential of human umbilical cord MSC‐derived EVs in both in vitro and in vivo models by upregulating miR‐135b‐5p and miR‐499a‐3p.^[^ ^57^ ^]^

Despite these promising results, the mechanisms by which lightwave and soundwave stimulation affect the therapeutic efficacy of EVs remain unclear and are currently the subject of ongoing investigation. Key factors, such as the optimal intensity of light and ultrasound power for maximizing EV efficacy, have yet to be determined. Additionally, there are concerns that these types of stimulation could inadvertently induce the secretion of unwanted components, such as apoptotic bodies, which may compromise the therapeutic effects of EVs due to the photothermal effect. Further research is needed to clarify these potential risks and optimize the use of light and sound waves in EV‐based therapies.

### 4.3 Biochemical Factors to Modulate EVs for Improved Therapeutic Efficacy

#### 4.3.1 Bioactive Molecules

It is well‐established that cells exposed to inflammatory stimuli, such as lipopolysaccharides (LPS) and inflammatory cytokines, can enhance their therapeutic efficacy.^[^ ^58^ ^]^ A growing body of evidence suggests that LPS and inflammatory cytokines, such as IFN‐γ, TNF‐α, and transforming growth factor‐beta 1 (TGF‐β1) can enhance the therapeutic potential of MSC‐derived EVs. These EVs have been shown to promote M2 macrophage polarization, reduce inflammation, and facilitate tissue repair in conditions such as osteoarthritis,^[^ ^59^ ^]^ wound healing^[^ ^60^ ^]^ and myocardial infarction.^[^ ^61^ ^]^ For instance, pretreatment of MSCs with IL‐1β has been shown to increase the secretion of EVs containing embedded miRNAs, which exhibit anti‐inflammatory and chondroprotective effects through key signaling pathways, including Nrf2, Wnt, Notch, TGFβ, and IHH, leading to improved outcomes in inflammatory conditions.^[^ ^62^ ^]^ Similarly, EVs derived from TNFα‐pretreated cells have demonstrated enhanced anti‐inflammatory and antifibrotic properties, showing therapeutic potential in conditions such as bone defect,^[^ ^63^ ^]^ urethral stricture,^[^ ^64^ ^]^ and acute liver failure.^[^ ^65^ ^]^

Growth factors, such as erythropoietin (EPO), bone morphogenetic protein 2 (BMP2), and TGFβ1, play crucial roles in modulating the recruitment, differentiation, and functional enhancement of MSCs.^[^ ^59^, ^66^ ^]^ These growth factors also prime MSCs to produce EVs that actively modulate inflammatory responses and support tissue repair.^[^ ^59c^ ^]^ For instance, BMP2 and TGFβ1 have been shown to enhance the efficacy of EVs, contributing to their regenerative potential by promoting bone regeneration and reducing cartilage damage in osteoarthritis.^[^ ^59c^ ^]^ Additionally, the synergistic interaction between multiple cytokines can lead to cumulative therapeutic effects. Zhang et al. found that the combined stimulation of MSCs with TGFβ1 and IFNγ produced EVs with superior immunosuppressive activity compared to stimulation by either cytokine alone.^[^ ^59d^ ^]^

In addition to growth factors, thrombin has been identified as a potent agent for enhancing EV biogenesis and cargo enrichment through protease‐activated receptor‐mediated signaling pathways.^[^ ^67^ ^]^ Beyond thrombin, EVs preconditioned by hormones such as melatonin can alleviate chronic kidney disease by upregulating miR‐4516 content in EVs,^[^ ^68^ ^]^ and improve diabetic wound healing by modulating macrophage polarization.^[^ ^69^ ^]^

#### 4.3.2 Small Molecule Compounds

**Figure 6:**
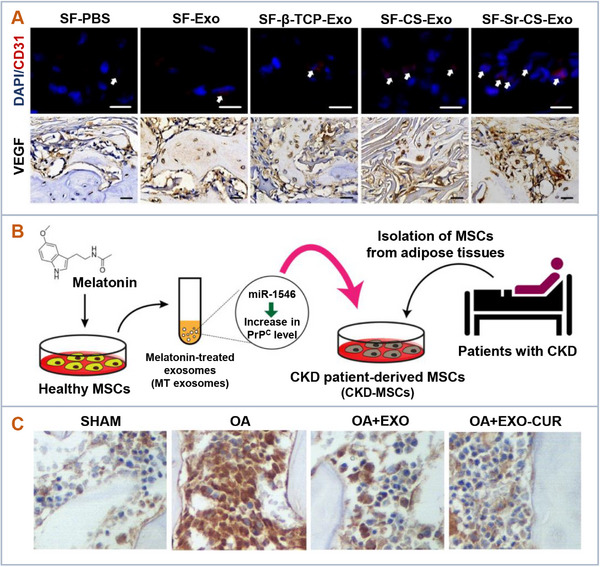
Biochemical stimulation to enhance therapeutic efficacy of extracellular vesicles. A) Strontium‐substituted calcium silicate ceramics enhanced the expression of miR‐146a in MSC‐derived EVs, which subsequently promoted the angiogenesis of implanted scaffolds. Adapted with permission.^[^ ^75a^ ^]^ Copyright 2021, Acta Biomaterialia. B) Melatonin treatment upregulated miR‐4516, leading to increased expression of cellular prion protein (PrPC) in EVs derived from MSCs. These EVs improved the proliferative and angiogenic potential of recipient cells. Adapted with permission.^[^ ^69^ ^]^ Copyright 2020, Journal of Pineal Research. C) Curcumin enhanced MSC‐derived EVs by modulating miR‐124 and miR‐143, effectively reducing the progression of osteoarthritis. Adapted with permission.^[^ ^72b^ ^]^ Copyright 2020, Journal of Cellular and Molecular Medicine.

Small molecule compounds are versatile tools in regenerative medicine due to their ability to influence cellular behavior.^[^ ^70^ ^]^ Dimethyloxaloylglycine (DMOG), a prolyl hydroxylase inhibitor, stabilizes hypoxia‐inducible factors (HIFs), thereby activating pathways linked to the cellular response to hypoxia.^[^ ^71^ ^]^ Liang et al. demonstrated that low doses of DMOG enhance the pro‐angiogenic activity of MSC‐derived EVs, promoting angiogenesis and bone regeneration through the activation of the AKT/mTOR signaling pathway.^[^ ^71^ ^]^ Other small molecules, such as curcumin or kartogenin, have also been shown to improve the efficacy and consistency of EVs, enhancing their therapeutic potential for cartilage regeneration and osteoarthritis treatment (**Figure** **6**).^[^ ^72^ ^]^ Atorvastatin, a statin known for its cholesterol‐lowering and pleiotropic effects, increased the pro‐angiogenic capacity of EVs derived from preconditioned donor cells in diabetic wound healing.^[^ ^73^ ^]^ Furthermore, lithium chloride boosted the neuroprotective effects of EVs by modulating miRNA content, leading to reduced post‐stroke inflammation and improved neuroregeneration and neurological recovery.^[^ ^74^ ^]^ To conclude, small molecules offer a scalable and cost‐effective alternative to more complex biological compounds, making them practical for large‐scale therapeutic applications. However, high concentrations of small molecules may pose toxicity risks, emphasizing the need for careful evaluation of their safety in clinical settings.

#### 4.3.3 Biochemical Compounds from Materials

Inorganic ceramic materials offer excellent biocompatibility and strong mechanical properties, making them promising candidates in regenerative medicine. Chen et al. showed that EVs derived from macrophages treated with biphasic calcium phosphate ceramics exhibited notable immunomodulatory, pro‐angiogenic, and pro‐osteogenic effects, shedding light on biomaterial‐mediated osteoinduction.^[^ ^20b^ ^]^ Similarly, EVs derived from MSCs and endothelial progenitor cells stimulated with silicate ceramics and nitric oxide‐releasing polymers promoted angiogenesis by upregulating miRNAs and VEGF, respectively.^[^ ^75^ ^]^ In addition to bulk materials, surface modifications can also significantly impact EV potency. Lee et al. found that simple modifications with short peptides, such as the glycine‐aspartic acid (RGD) sequence, substantially enhanced the pro‐survival effects of EVs secreted by human induced pluripotent stem cells.^[^ ^76^ ^]^

In summary, these studies collectively highlight the importance of designing tailored microenvironments for stem cell culture to optimize EVs composition and potency, thereby advancing the development of cell‐free therapeutic applications. However, the materials and biomolecules used must comply with FDA regulations to ensure that the EVs generated from cells exposed to these biochemical factors are safe for subsequent applications. To ensure this, the concentrations of the stimulating compounds within the EVs need to be precisely measured and analyzed to confirm that they do not present any safety risks. Additionally, it is crucial to recognize that the optimal concentration of certain molecules that are effective in one cell line may not be effective in another.

## 5 Limitations and Future Perspectives

Addressing the therapeutic efficacy is essential for advancing the clinical application of EVs in disease treatment. Although significant progress has been made in understanding how the microenvironment affects the therapeutic efficacy of EVs, many critical issues remain unexplored. One of the main challenges in clinical applications is to maintain the quality and consistency of extracellular vesicles during large‐scale production for their therapeutic applications. Aiming this, standardization is required at multiple stages, including EV production, isolation, characterization, and storage. For instance, well‐characterized and homogeneous cell lines or primary cells with controlled passage numbers, along with the use of bioreactors instead of traditional 2D Petri dishes, can facilitate large‐scale, reproducible EV production while minimizing variability.^[^ ^77^ ^]^ Throughout this process, maintaining uniform oxygen levels, pH, temperature, and nutrient supply is essential to reduce batch‐to‐batch variability and ensure EV reproducibility. Additionally, comprehensive EV characterization is necessary to confirm that scalable EVs meet defined quality standards, including morphology, size distribution, concentration, purity, specific markers, and cargo content. Furthermore, proper storage conditions are crucial to prevent EV degradation. Storing EVs at −80 °C or utilizing lyophilization for long‐term preservation can help maintain their stability and quality for therapeutic use.^[^ ^78^ ^]^ In essence, achieving EV quality consistency requires strict adherence to Good Manufacturing Practice (GMP) guidelines and the implementation of Standard Operating Procedures (SOPs) throughout the production and characterization process.

Another key concern in the clinical use of EVs is their potential immunogenicity. Although their toxicity is low due to their similarity to naturally occurring biological materials, EVs derived from xenogeneic or allogeneic sources are more likely to undergo enhanced clearance by immune systems.^[^ ^79^ ^]^ To reduce the immunogenicity of EVs in therapeutic applications, surface engineering, genetic modifications, and optimized production methods are being explored to minimize immune recognition, such as the addition or removal of specific surface components,^[^ ^80^ ^]^ as well as the engineering of immunoevasive surfaces.^[^ ^79^ ^]^ An alternative approach is in situ stimulation, where the source cells are directly stimulated within their native environment to produce EVs with enhanced therapeutic properties. For instance, in situ, EV production can be precisely controlled and enhanced using superparamagnetic and mechanoelectronic stimulation.^[^ ^55^, ^79^ ^]^ In the future, specific stimuli may be applied directly at the treatment sites, enabling tissues or cells to generate highly effective EVs for repair while minimizing immunotoxicity.

Current research predominantly focuses on the effects of EVs on recipient cells, while the mechanisms governing EV biogenesis remain poorly understood. This gap underscores the need for future studies to investigate the processes involved in EV formation and release, as such knowledge could provide key insights into optimizing both the production and functionality of EVs for therapeutic purposes. Additionally, the cargo contents of EVs are recognized as the main mediators of EVs for regenerative capacities. However, key factors and mechanisms underlying these effects in different diseases are yet to be identified. The application of omics technologies, such as proteomics, transcriptomic, single‐cell, and exosome sequencing, holds significant potential for uncovering and characterizing these critical components.

Except for fundamental research that explores the mechanism, several innovative strategies can be employed to enhance EV therapeutic efficacy. A second strategy involves applying multiple stimuli, either simultaneously or sequentially, to synergistically increase EV production and efficacy. For instance, Patel et al. showed that the combination of a 3D‐printed scaffold‐perfusion bioreactor system with ethanol conditioning significantly improved both the yield and therapeutic potency of endothelial cell‐derived EVs.^[^ ^81^ ^]^ Similarly, dynamic culture systems that pair hypoxia with chemical conditioning, such as ethanol or interferon‐gamma (IFNγ), have been shown to substantially increase EV production and enhance their bioactivity.^[^ ^12^, ^82^ ^]^ The other strategy centers on post‐translational modifications, allowing for the engineering of EVs to improve their stability, targeting capabilities, or bioactivity. This approach enables the control of gene expression without altering the DNA sequence, offering a safer alternative for enhancing EV therapeutic efficacy by modifying the parental cells.^[^ ^83^ ^]^ Finally, the selection of a specific, standardized cell type for EV production could offer substantial benefits for broader clinical applications. Many parent cells are difficult to obtain, and their EV yields are often low and inconsistent. Identifying an easily accessible, non‐invasive cell source capable of producing high‐quality EVs could streamline the production process. By precisely controlling the microenvironmental parameters for this standard cell type, it could be engineered to produce a wide array of EVs with tailored therapeutic properties. This approach would not only simplify the manufacturing process but also reduce the complexity and cost of developing cell‐specific therapies.

## 6 Conclusion

In conclusion, there is an expanding body of research investigating ways to enhance the therapeutic efficacy of EVs by modulating the microenvironment signals, including physical, mechanical, and biochemical signals. Nevertheless, substantial gaps remain, particularly regarding the mechanisms underlying EV biogenesis and the factors that influence their therapeutic efficacy. Innovative strategies, including in situ stimulation, the use of synergistic stimuli, post‐translational modifications, and the standardization of specific cell types, present promising opportunities for addressing these challenges. These approaches ultimately hold the potential to advance the development of effective EV‐based therapies for clinical use.

## Conflict of Interest

The authors declare no conflict of interest.

## Author Contributions

B.X. and Y.Z. gave the concept and designed the study. B.X., S.Z., X.G., and Y.Z. conducted the literature review. All authors wrote the manuscript and commented on the manuscript.
